# First-generation *EGFR* tyrosine kinase inhibitor therapy in 106 patients with compound *EGFR*-mutated lung cancer: a single institution’s clinical practice experience

**DOI:** 10.1186/s40880-018-0321-0

**Published:** 2018-07-28

**Authors:** Xiangyang Yu, Xuewen Zhang, Zichen Zhang, Yongbin Lin, Yingsheng Wen, Yongqiang Chen, Weidong Wang, Lanjun Zhang

**Affiliations:** 1 State Key Laboratory of Oncology in South China, Collaborative Innovation Center for Cancer Medicine, Guangzhou, 510060 Guangdong China; 2 Department of Thoracic Surgery, Sun Yat-sen University Cancer Center, 651 Dongfeng Road East, Guangzhou, 510060 Guangdong China; 3 Department of Medical Oncology, Sun Yat-sen University Cancer Center, Guangzhou, 510060 Guangdong China; 4 Department of Molecular Pathology, Sun Yat-sen University Cancer Center, Guangzhou, 510060 Guangdong China

**Keywords:** *EGFR*, TKIs, Compound mutations

## Abstract

**Background:**

The antitumour efficacy of tyrosine kinase inhibitors (TKIs) in lung cancer patients with compound epidermal growth factor receptor (*EGFR*) mutations has not been resolved. Our study summarizes a single institutional experience of first-generation TKI therapy for lung cancers with compound *EGFR* mutations.

**Methods:**

A total of 106 consecutive patients with tumours bearing compound *EGFR* mutations were identified between January 2012 and May 2016; all patients received first-generation TKI therapy. Deletions in exon 19 and the *L858R* point mutation in exon 21 were considered common mutations; *T790M* was considered separately because of its association with TKIs resistances. Any other mutation was defined as a rare mutation. Patients were divided as follows: double common mutations (group A); common plus *T790M* mutations (group B); common plus rare mutations (group C); double rare mutations (group D); and rare plus *T790M* mutations (group E). A separate group of 115 consecutive patients with a single common mutation was created for comparative analysis (group F).

**Results:**

The frequency of patients with compound *EGFR* was 2.9% (114/3925) and their response rate to first-generation TKIs was 50.9%, which was not significantly different from group F (67.0%, *P *= 0.088). The progression-free survival (PFS) of the 106 patients receiving TKI therapy was worse than that of group F (median, 9.1 vs. 13.0 months, respectively; *P *< 0.001). The PFS of the compound mutation group was shorter than that of the single common mutation group (median, 10.1 months in group A, *P *= 0.240; 9.1 months in group B, *P *< 0.001; 9.6 months in group C, *P* = 0.010; 6.5 months in group D, *P *= 0.048; 5.4 months in group E, *P *= 0.017). Patients with a co-occurring mutation in exon 20 (excluding T790M) exhibited significantly worse PFS than the patients with other compound mutations or with a single common mutation (median, 6.5 vs. 9.1 vs. 13.0 months, respectively, *P *= 0.002).

**Conclusions:**

There was significant heterogeneity among the compound *EGFR* mutations and their response to first-generation TKIs. Individualized treatment in clinical practice should be considered for each case.

## Introduction

Lung cancer continues to be the primary cause of cancer death both in China and worldwide. The 5-year survival rate for lung cancer has increased from 12% in the 1970s to 17.7% in 2016 [1], and these sufferers primarily include numerous patients with advanced lung cancer. Many advances have been made in the treatment of advanced lung cancer, especially with regard to targeted therapies, and these treatment strategies have resulted in considerable improvements in survival. Among them, the receptor tyrosine kinases (RTKs) super-family of cell surface receptors serve as mediators of cell signaling by extra-cellular growth factors. Members of the ErbB family of RTKs, such as ErbB1 (also known as *EGFR*), ErbB2, ErbB3 and ErbB4, have received much attention, given their strong association with malignant proliferation [2, 3].

Over the past decade, three small-molecule ErbB tyrosine kinases inhibitors (TKIs) have been shown to efficiently target tumour cell survival pathways in advanced non-small cell lung cancers (NSCLC) expressing the epidermal growth factor receptor (*EGFR*): gefitinib (approved by the US Food and Drug Administration in May 2003), erlotinib (approved by the US Food and Drug Administration in November 2004) and icotinib (approved by China’s State Food and Drug Administration in June 2011). The use of these agents has resulted in higher overall response rates (ORRs, up to 60%–70%) and longer progression-free survival (PFS; 9–16 months) and overall survival (OS; exceeding 20 months) than current first-line platinum-based chemotherapies [1–5].

These patients with classical *EGFR* genetic mutations that are sensitive to TKIs involve in-frame deletions of exon 19 and L858R substitutions of exon 21 and occur in approximately 85%–90% of all *EGFR*-mutated patients [1, 4–6]. However, compound *EGFR* mutations with or without classical mutations have been detected within the same tumour tissues in some patients [7–14]. Previous studies reported that only 2%–15% of the population with *EGFR* mutations exhibits these rare compound mutations [7–14]. The characteristics of this rare population, the efficacy of *EGFR* TKIs, and the prognostic value of the compound mutations have not been clarified because of the very low rate of these mutations. Furthermore, the lack of adequate evidence-based medical research hinders treatment decisions when these co-existing double-site mutants are detected.

The present cohort analysis examined two major questions: first, we investigated the incidence of different compound *EGFR* mutation subtypes in a single institution; and second, we investigated this population’s characteristics and the efficacy (ORRs and PFS) of first-generation small-molecule TKI treatment and prognosis compared with patients bearing the classical mutation alone.

## Materials and methods

### Patient selection

We retrospectively reviewed a molecular diagnostic database of lung cancer in the Sun Yat-sen University Cancer Center (SYSUCC) between January 2012 and May 2016. The database was screened for *EGFR*-mutated cases, and a cohort of consecutive cases with compound *EGFR* mutations was identified. The co-existence of two different *EGFR* mutation sites detected in a single tumour specimen was defined as a compound *EGFR* mutation. The hospital’s ethics committee approved the research using this micro-database, and all subjects provided written informed consent.

Patients were eligible for enrolment if they had received daily oral *EGFR* TKIs, such as gefitinib (250 mg, qd), erlotinib (150 mg, qd) or icotinib (125 mg, tid), until disease progression or death; complete follow-up information was obtained from the medical record department. The co-existence of compound mutations with *T790M* must have been detected prior to initiation of targeted therapy.

Exclusion criteria included second primary neoplasms diagnosed before/after the lung cancer, intolerant levels of toxicity, loss to follow-up, and refusal of treatment.

### Sequencing of EGFR mutations in exons 18–21

Details of the genetic sequencing and molecular analysis were described previously [6]. Briefly, tumour cell DNA was extracted from paraffin-embedded specimens that contained at least 50% tumour cells, including surgically resected tumour specimens, fine needle aspiration biopsies of lymph nodes or metastatic lesions, and tumour cells from pleural fluid, using a QIAamp DNA FFPE Tissue Kit (Qiagen, Hilden, Germany) in strict accordance with the manufacturer’s recommendations. The tumour cell DNA was examined for *EGFR* mutation(s) in exons 18–21, including 36 TKI-responsive mutation sites and nine TKI-resistant mutation sites, using an amplification refractory mutation system polymerase chain reaction (ARMS-PCR) kit (GP Medical Technology Co. Ltd., Beijing, China) [6]. Mass ARRAY TYPER 4.0 software (Sequenom Inc., San Diego, CA, USA) was used to individually classify *EGFR*-positive mutation sites when the mutation frequency was higher than 1%.

### Follow-up and end-point

A systemic baseline assessment, including chest and abdomen enhanced computed tomography (CT) scanning and brain enhanced magnetic resonance imaging examination, was routinely performed prior to *EGFR* TKI treatments for locally advanced, recurrent, or metastatic lung cancer. A follow-up assessment was generally performed every 3 months after the 1st day of TKI treatment until February 28, 2017, radiographic progression or death. Two board-certified radiologists independently evaluated the therapeutic effectiveness based on the Response Evaluation Criteria in Solid Tumours (RECIST) and classified the therapeutic effect into four levels: progressive disease (PD), stable disease (SD), partial response (PR), and complete response (CR). Patients exhibiting PD or SD were considered non-responders to targeted treatments, while patients with PR or CR were regarded as effective disease control by antitumour agents. Follow-up information was extracted from the patients’ complete medical and radiological records.

The duration of PFS was calculated from the 1st day of *EGFR* TKI treatment to the last follow-up, the date of death or when disease progression was first observed. The duration of OS was also evaluated from the date of the 1st day of *EGFR* TKI treatment until the last follow-up or the date of death from any cause. Patients were censored at their last known progression-free or alive date.

### Data analysis

Complete medical, pathological and radiological data and molecular diagnostics were analysed using the Statistical Package for the Social Sciences for Windows version 23.0 (SPSS Inc., Chicago, IL, USA). Categorical variables were compared between the *EGFR* mutant subgroups (i.e., single common mutation, double common mutations, common plus *T790M* mutations, common plus rare mutations, rare plus rare mutations, and rare plus *T790M* mutations) using Chi square (χ^2^) and Fisher’s exact tests. The Kaplan–Meier method was used to construct the PFS and OS curves of each subgroup, and significant differences between survival curves were examined using the log-rank test. A two-sided *P* value less than 0.05 was used to confirm significant differences.

## Results

### Clinical and pathological features of the population with compound *EGFR* mutations

**Fig. 1:**
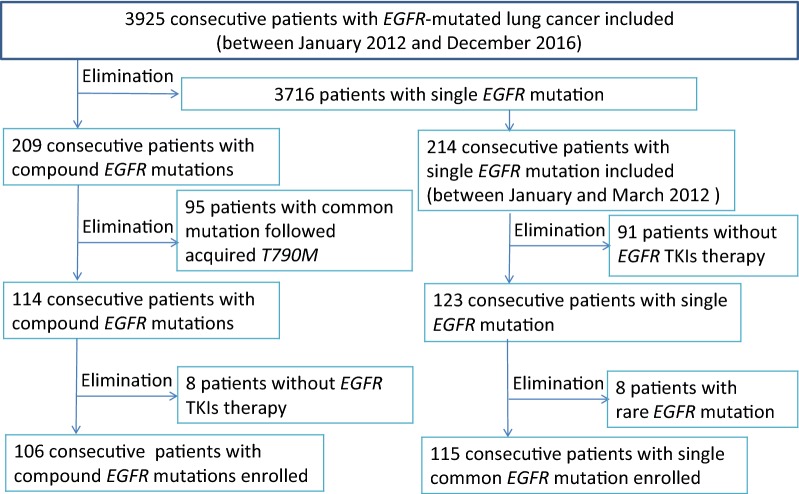
Screening procedure of the 106 lung cancer patients with compound *EGFR* mutations

A total of 3925 NSCLC patients with *EGFR* mutations were identified using ARMS-PCR between January 2012 and December 2016 in SYSUCC. A total of 209 consecutive patients with compound *EGFR* mutations were identified among the 3925 NSCLC patients. However, 95 cases with the *19Del* or *L858R* mutations that acquired the *T790M* after TKI therapy and 8 cases that did not receive TKI therapy in our hospital were excluded. Only 106 patients (2.9%) with primary co-existing double *EGFR* mutations received first-generation TKI therapy and were entered into our analysis (Fig. 1).

Most of the 106 patients were non-geriatric (63/106, 59.4%; median age at initial diagnosis, 57 ± 10.7 years), non-smokers (69/106, 65.1%) and had advanced lung cancer (86/106, 81.1%) (Table 1). All initial pathological stage I -IIIA patients (20/106, 18.9%) received radical resection. No sex predilection (55 women and 51 men) was present in our cohort. Notably, a compound *EGFR* mutation was detected in four non-adenocarcinoma patients, including two squamous cell carcinomas (SCC, *L858R *+ *L858Q* and *L858R *+ *T790M*), one lymphoepithelioma-like carcinoma (LELC, *19Del *+ *L858R*) and one adenosquamous carcinoma (ASC, *19Del *+ *T790M*). The first-generation *EGFR* TKIs were primarily administered as first (55/106, 51.9%) or second line (46/106, 43.4%) treatment.

**Table 1:** Clinicopathological characteristics of lung cancer patients with compound *EGFR* mutations

Variable	Single common mutation (n = 115)	Compound mutations (n = 106)	*P* ^†^	Double common mutations (n = 5)	*P* ^†^	Common + rare mutations (n = 11)	*P* ^†^	Rare + rare mutations (n = 13)	*P* ^†^	Rare + *T790M* mutations (n = 8)	*P* ^†^	Common + *T790M* mutations (n = 69)	*P* ^†^
Sex													
Female	56	55	0.635	1	0.368	6	0.711	6	0.862	4	0.943	38	0.402
Male	59	51		4		5		7		4		31	
Age (years)													
< 60	57	63	0.141	2	0.675	5	0.794	8	0.413	7	0.063	41	0.195
≥ 60	58	43		3		6		5		1		28	
Smoking status			0.270		0.118		0.795		0.442		0.795		0.022
Non-smoker	69	69		1		5		4		5		54	
Smoker	34	24		3		3		4		3		11	
Unknown	12	13		1		0		5		0		4	
Tumour status			0.034		0.592		0.782		0.753		0.103		0.064
Recurrence	36	20		1		3		3		0		13	
Initial IIIb–IV	79	86		4		8		10		8		56	
ECOG PS													
0–1	107	96	0.501	4	0.327	9	0.212	13	0.326	6	0.071	64	0.941
2–4	8	10		1		2		0		2		5	
Pathology			0.529		0.141		0.672		0.899		0.948		0.534
ADC	110	102		4		10		13		8		67	
SCC	5	2		0		1		0		0		1	
LELC	0	1		1		0		0		0		0	
ASC	0	1		0		0		0		0		1	
Timing of TKI			0.720		0.914		0.835		0.803		0.805		0.460
First line	52	55		2		5		6		3		39	
Second line	55	46		3		6		7		5		25	
Third line	7	4		0		0		0		0		4	
Fourth line	1	1		0		0		0		0		1	
TKI selection			0.034		0.981		0.592		0.214		0.161		0.021
Gefitinib	64	47		3		5		4		2		33	
Erlotinib	26	41		1		4		4		4		28	
Icotinib	25	18		1		2		5		2		8	
Response to TKIs													
PD	10	14	0.088	1	0.148	2	0.652	2	0.328	4	0.012	5	0.293
SD	21	33		1		3		5		1		22	
PR	77	54		3		5		5		3		39	
CR	1	0		0		0		0		0		0	
NE	6	5		0		1		1		0		3	

*ECOG PS* Eastern Cooperative Oncology Group performance status, *ADC* adenocarcinoma, *SCC* squamous cell carcinoma, *LELC* lymphoepithelioma-like carcinoma, *ASC* adenosquamous carcinoma, *PD* progressive disease, *SD* stable disease, *PR* partial response, *CR* complete response, *NE* not evaluated, *TKI* tyrosine kinase inhibitor, *PFS* progression-free survival, *OS* overall survival

^**†**^All variables of different subgroups were compared with the single common mutation group; *P* < 0.05 was defined as significantly different

A total of 115 consecutive patients with a single common mutation (*19Del* or *L858R*) who received first-generation *EGFR* TKI therapy between January and March 2012 were selected for comparative analysis (Fig. 1).

### Distribution frequency of compound *EGFR* mutations

We divided the cohort with the compound *EGFR* mutations into five groups based on the categories of common and rare mutations sites reported in the literature: a double common mutation group (5/106, 4.7%), a common plus rare mutation group (11/106, 10.4%), a common plus *T790M* mutation group (69/106, 65.1%), a double rare mutation group (13/106, 12.3%), and a rare plus *T790M* mutation group (8/106, 7.5%) (Table 1) [3]. The most frequent mutation site was *T790M* (77/106, 72.6%), and the majority of patients harboured *19Del* (50/106, 47.2%) or *L858R* (40/106, 37.7%) as one of the compound mutations (Table 3). Notably, the most frequent compound mutation involved a common mutation co-existing with *T790M* (69/106, 65.1%), and these common mutations included *19Del* or *L858R* with *T790M*. The most frequent uncommon mutation was *L858Q* (13/106, 12.3%), followed by *S768I* (9/106, 8.5%), *G719X* (7/106, 6.6%), *G719S* (5/106, 4.7%), *D761Y* (2/106, 1.9%), *S720P* (1/106, 0.9%), *K757R* (1/106, 0.9%), *I744M* (1/106, 0.9%), *R776C* (1/106, 0.9%), *L833V* (1/106, 0.9%), *E709A* (1/106, 0.9%), and *V774M* (1/106, 0.9%).

### PFS and patient response after TKI treatment

**Fig. 2:**
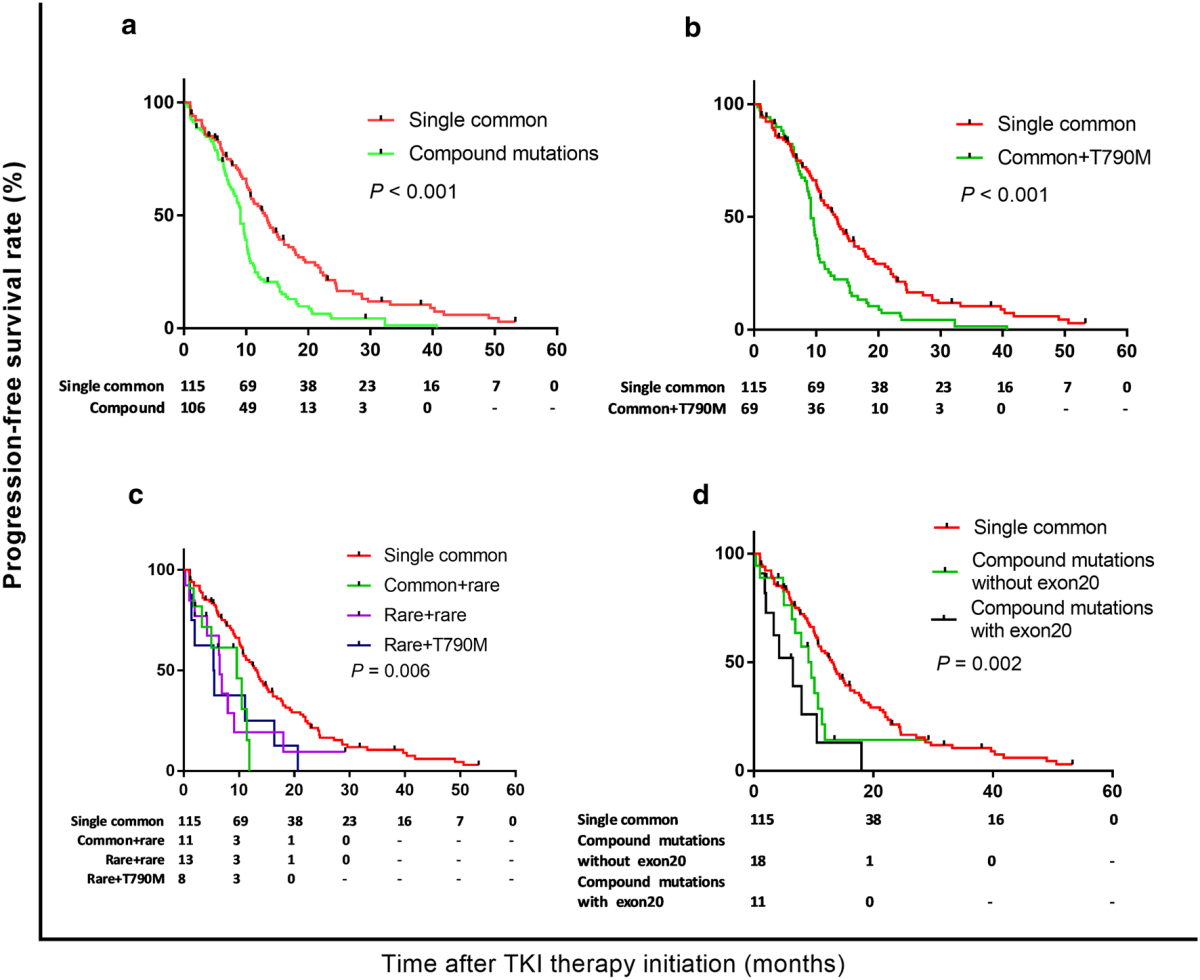
Progression-free survival by mutation status: **a** a single common *EGFR* mutation vs. compound *EGFR* mutations (median PFS: 9.1 vs. 13.0 months, respectively; *P *< 0.001), **b** a single common *EGFR* mutation vs. common + *T790M* mutations (median: 9.1 months vs. 13.0 months, *P* < 0.001), **c** a single common *EGFR* mutation vs. common + rare mutations vs. rare + rare mutations vs. rare + *T790M* mutations (median: 13.0 months vs. 10.5 months vs. 6.5 months vs. 5.4 months, *P* = 0.006), and **d** a single *EGFR* mutation vs. compound mutations without exon 20 vs. compound mutations with exon 20 (median: 13.0 months vs. 9.1 months vs. 6.5 months, *P* = 0.002)

The median follow-up time in the compound *EGFR* mutation cohort was 29.4 months (range, 1.5–119.5 months), and the 1-, 2-, and 3-year PFS rates after *EGFR* TKI treatment were 32.7, 4.3, and 1.4%, respectively, which were all significantly lower than the population with a single common mutation (1-, 2-, and 3-year PFS rates were 54.1, 20.1, and 10.5%, respectively, *P *< 0.001) (Fig. 2a). Twenty-six tumour-related deaths occurred during follow-up, and the median OS was not reached for all patients with compound mutations. Univariate analysis of the total 221 patients who received first-generation TKI therapy revealed that compound mutations were significantly correlated with shorter duration of targeted therapy (HR: 1.883, 95% *CI* 1.404–2.526, *P *< 0.001), in addition to initial advanced status, non-adenocarcinoma, and more than second-line treatment (Table 2). Inclusion of these variables in the multivariate analysis revealed that these four factors were also independent significant PFS factors.

**Table 2:** Univariate and multivariate analysis for progression-free survival to first-generation TKI therapy

	Univariate analysis		Multivariate analysis	
	HR (95% CI)	*P* ^†^	HR (95% CI)	*P* ^†^
Sex				
(Female/male)	1.061 (0.919–1.224)	0.420		
Age				
(< 60/≥ 60)	1.319 (0.987–1.763)	0.061		
Smoking status				
(Smoker/nonsmoker/unknown)	0.726 (0.518–1.018)	0.063		
Tumour status				
(Recurrence/initial IIIb–IV)	0.721 (0.560–0.926)	0.011	0.706 (0.548–0.909)	0.007
ECOG PS				
(0–1/2–4)	1.438 (0.872–2.372)	0.154		
Pathology				
(Non-adeno/adeno)	4.175 (2.113–8.250)	0.001	5.472 (2.623–11.417)	0.001
Timing of TKI				
(1/≥ 2)	0.643 (0.480–0.862)	0.003	0.610 (0.452–0.823)	0.001
*EGFR* mutation status				
(Compound/single)	1.883 (1.404–2.526)	0.001	1.981 (1.466–2.676)	0.001
TKI selection				
(Gefitinib/erlotinib/icotinib)	0.978 (0.819–1.167)	0.806		

*ECOG PS* Eastern Cooperative Oncology Group performance status, *HR* hazard ratio, *CI* confidence interval

^**†**^All variables of different subgroups were compared with the single common mutation group; *P* < 0.05 was defined as significantly different

Among the five patients with double common *EGFR* mutations (*19Del* plus *L858R*), only one stage IV patient with brain metastases exhibited effective local control, i.e., SD in response to gefitinib, but progression of the primary pulmonary neoplasm was identified after 7.9 months. Three other patients with advanced lung cancer exhibited a PR to oral gefitinib or icotinib therapy, and their prolonged PFS times was longer than 10 months (10.1, 10.7 and 13.5 months, respectively) (Table 3). Notably, the patient with primary pulmonary LELC, whose tumour harboured a *19Del* plus *L858R* mutation, was diagnosed at stage IV because of osseous metastasis. However, no antitumour activity of erlotinib against this rare subtype of lung cancer was observed, i.e., there was PD. There were no significant differences in the response rates (RR, 25.0% vs. 67.0%, *P* = 0.908, using Chi square tests) or PFS (median, 10.1 months vs. 13.0 months, *P *= 0.240, using log-rank tests) compared with those of the group with a single common mutation.

**Table 3:** Frequency, detailed combination patterns, progression-free survival, overall survival and response to first-generation TKIs of compound *EGFR* mutations

Subgroup of compound EGFR mutations	Frequency (n, %)	Mutated exons	Response (rate, %)	PFS (range, months)	OS (range, months)
Double common	5 (4.7)		25.0%	10.1 ± 2.4	24.2 ± 8.2
*19Del *+* L858R*	5	19 and 21	3PR, 1SD, 1PD	4.9–12	13.1–25.6
Common + rare	11 (10.4)		45.5%	10.5 ± 3.9	Not reached
*19Del *+* L861Q*	2	19 and 21	1PR, 1SD	11.9–14.4	26.5–41.2
*L858R *+* S720P*	1	21 and 18	PD	2.1	2.1
*L858R *+* K757R*	1	21 and 19	PR	9.0	8.7
*L858R *+* I744* *M*	1	21 and 19	PR	17.6	41.2
*L858R* + *S768I*	3	21 and 20	1PR, 1PD, 1SD	1.8–6.2	4.0–12.5
*L858R *+* R776H*	1	21 and 20	PR	10.5	12.6
*L858R *+* L858Q*	1	21 and 21	NE	1	3
*L858R *+* L833V*	1	21 and 21	SD	5.0	15.9
Common + *T790M*	69 (65.1)		56.5%	10.3 ± 0.6	Not reached
*19Del *+* T790M*	43	19 and 20	27PR, 12SD, 2 PD, 2NE	0.6–40.7	0.2–88.5
*L858R *+* T790M*	26	21 and 20	12PR, 10SD, 3PD, 1NE	0.9–24.1	1.2–56.6
Rare + rare	13 (12.3)		38.5%	6.5 ± 1.3	Not reached
*G719C *+* S768I*	1	18 and 20	PR	6.5	13.2
*G719S *+* S768I*	2	18 and 20	1PR, 1SD	1–8.0	2.0–8.4
*G719S *+* L858Q*	1	18 and 21	SD	6.4	29.3
*G719X *+* S768I*	3	18 and 20	2PR, 1SD	2.0–18	2.0–44.0
*G719X* + *L858Q*	3	18 and 21	1SD, 1PD, 1NE	0.3–27.3	2.3–29.2
*G719S* +* E709A*	1	18 and 18	PR	4.1	4.1
*G719S *+* L858Q*	1	18 and 21	SD	6.4	29.3
*S768I *+* V774M*	1	20 and 20	PD	2.0	13.8
Rare + *T790M*	8 (7.5%)		37.5%	5.4 ± 2.5	23.8 ± 1.5
*G719X* +* T790M*	1	18 and 20	PR	11.1	55.6
*D761Y* + *T790M*	2	19 and 20	2PD	1.1–5.5	1.1–8.5
*L858Q *+* T790M*	5	21 and 20	2PR, 1SD, 2PD	1.4–20.6	18–88.3

*TKI* tyrosine kinase inhibitor, *PFS* progression-free survival, *OS* overall survival, *PR* partial response, *SD* stable disease, *CR* complete response, *PD* progressive disease, *NE* not evaluated

The RR to TKI in the patients with common mutations (*19Del* or *L858R*) plus *T790M*, which was not a mutation acquired during oral *EGFR* TKI, was 56.5% (39/69); this rate was not significantly different than that of patients with a single *19Del* or *L858R* mutation (*P* = 0.293) (Table 1). These 69 patients exhibited a worse PFS than the 115 patients with a primary *T790M* (median, 9.1 months vs. 13.0 months, respectively; *P *< 0.001) (Fig. 2b). Approximately 33% of the patients (23/69) were enrolled in an AZD9291 international multicentre, single-arm phase 2 clinical trial after progression was detected using CT scanning during treatment with first-generation *EGFR* TKIs.

The compound *EGFR*-mutated patients with rare site involvement exhibited a lower RR (37.5% vs. 67.8%, *P *= 0.023) and a shorter median PFS than the single common mutation subgroup (median, 13.0 months vs. 6.5 months, respectively; *P *< 0.001) (Fig. 2c). However, there was no difference in PFS across the common plus rare mutations subgroup, double rare mutations subgroup, or the rare plus *T790M* mutations subgroup (median, 10.5 months vs. 6.5 months vs. 5.4 months, respectively; *P* = 0.984) (Table 1). The co-occurrence of mutations in exon 20 (excluding *T790M*) had a significant effect on PFS, which was worse than the other compound mutations and the single common mutation patients (median, 13.0 months vs. 9.1 months vs. 6.5 months, respectively; *P *= 0.002) (Fig. 2d).

## Discussion

Our single institution study identified 114 patients with compound *EGFR* mutations among 3925 patients with *EGFR* mutations (114/3925, 2.9%). The RR of this rare population to first-generation TKI therapy was 50.9%, which was lower than that of patients with a single common mutation, but the difference was not significant (*P* = 0.088). Patients with compound mutations exhibited a shorter duration of first-generation TKI therapy in multivariate analysis than patients with a single common mutation (HR, 1.981; 95% confidence interval (*CI*) 1.466–2.676; *P* < 0.001). Exclusion of patients with a co-occurrence of mutations in exon 20 (excluding *T790M*) revealed that the duration of targeted TKI therapy was even shorter for other types of compound mutations (*P* = 0.002).

The phenomenon of lung cancer cells harbouring multiple *EGFR* mutations is worth mentioning, and it reportedly has accompanied the clinical use of first-generation small-molecule TKIs since 2004 [7]. Most published studies on multiple mutations were case reports because the techniques for detecting *EGFR* mutations were used only to detect drug-sensitive mutations in exon 19 and 21, and some patients with compound mutations were likely undetected. Developments in mutational detection and analysis techniques, such as direct sequencing, multiplex PCR systems and next-generation sequencing, increased the number of reported cases with compound mutations between 2004 and 2017 (Table 4). The reported frequency of the rare population with compound mutations ranged from 2.6% to 15% [8–15], which was slightly higher than that observed in our cohort (2.9%).

**Table 4:** Literature review of patients harbouring compound *EGFR* mutations and PFS and response to first-generation TKIs between 2004 and 2017

Compound mutations	Double common (n, mPFS, response)	Common + rare (n, mPFS, response)	Rare + rare (n, mPFS, response)	Common + *T790M* (n, mPFS, response)	Rare + *T790M* (n, mPFS, response)
Kobayashi et al. [8]	None	3; 3 months^a^; 2 PR, 1 PD	4; 8 months; 4 PR	None	None
Zhang et al. [9]	3; 17.5 months; 1 CR, 1 PR, 1 NA	None	None	None	None
Hsieh et al. [10]	None	1; 1.9 months; 1 SR	6; 11.6 months; 4 PR, 2 PD	None	None
Hata et al. [11]	8; 12.7 months; 1 CR, 5 PR, 1 SD, 1 NA	8; 2.5 months; 2 PR, 1 SD, 2 PD, 3 NA	None	None	None
Keam et al. [14]	None	16; 8.1 months; 11 PR, 4 SD, 1 PD	3; 4.6 months; 1 PR, 1 PD, 1 NA	5; 8.0 months; 4 PR, 1 PD	None
Xu et al. [16]	14; 9.53 months; 10 PR, 3 SD, 1 PD	18; 9.8 months; 10 PR, 5 SD, 3 PD	None	9; 1.9 months; 2 PR, 3 SD, 4 PD	None
Wu et al. [17]	None	7; NA; 2 PR, 1 SD, 4 PD	3; NA; 2 PR, 1 PD	None	None
Chen et al. [18]	None	10; 8.9 months; 4 PR, 6 NA	None	3; 6.7 months; 1 PR, 1 SD, 1 NA	1; 6 months; SD
Wu et al. [21]	None	12; 13.5 months; 10 PR, 1 SD, 1 PD	7; 4.2 months; 2 PR, 4 SD, 1 PD	2; NA; 2 PR	None
Asahina et al. [26]	None	None	1; 1.1 months; PD	None	None
Zhang et al. [32]	2; 6.1 months; 2PR	7; NA; NA	11; NA; NA	8; 3.3 months; 1PR, 1SD, 6NA	3; NA; NA
Zhu et al. [33]	None	3; 5.3 months^a^; 2SD, 1PD	5; 3.5 months^a^; 2 PR, 2 SD, 1 NA	None	None
Wu et al. [34]	None	9; 8.6 months; 7 PR, 1 SD, 1 PD	4; 9.2 months; 2 PR, 1 PD, 1 SD	None	None
Yang et al. [35]	1; 2 months; 1 PD	None	None	None	None
Svaton et al. [36]	None	None	1; 8 months; 1 PR	None	None
Peng et al. [37]	2; 11.5 months; 1 PR, 1 SD	3; 10 months; 3 SD	None	1; 10 months; 1 SD	None
Baek et al. [38]	12; 7.4 months; 4 CR, 5 PR, 2 SD, 1 PD	None	11; 5.1 months; 5 CR, 4 PR, 2 SD	None	None
Peng et al. [39]	2; 11.5 months; 1 PR, 1 SD	4; 8 months; 4 SD	3; 3 months^a^; 1 CR, 1 SD	2; 9 months; 2 SD	None
Chung et al. [40]	None	1; 5 months^a^; PR	None	None	None
Yang et al. [41]	None	3; NA; 1 PR, 1 SD, 1 PD	2; NA; 2 SD	1; NA; PD	None
Ichihara et al. [42]	None	2; 2.4 months; 2 SD	None	1; 1.6 months; SD	None
Pugh et al. [43]	None	1; NA; PR	1; NA; PR	None	None
Kimura et al. [44]	None	1; 5 months; PR	None	None	None
Van Zandwijk et al. [45]	None	None	1; NA; PR	None	None
Jackman et al. [46]	None	1; 14.8 months^a^; SD	None	None	None
Pallis et al. [47]	None	3; NA; 1 PR, 1 SD, 1 PD	1; NA; PD	None	None
Han et al. [48]	None	1; 13.8 months; 1 SD	2; 3 months; 2 PR	None	None
Kosakaet al. [49]	None	2; 24.5 months; 2 PR	None	None	None
Choong et al. [50]	None	1; 8 months; PR	None	None	None
Oshita et al. [51]	None	2; 13.2 months; 2 PR	1; 12 months; SD	None	None
Tokumo et al. [52]	None	1; 2 months; PD	None	None	None
Chou et al. [53]	None	None	2; 4.1 months; 1 PD, 1 PD	None	None
Shih et al. [54]	None	2; NA; 2 PR	2; NA; 2 PR	None	None
Taron et al. [55]	None	1; 9.4 months; PR	None	None	None
Mitsudomi et al. [56]	None	1, NA,1 PD	None	None	None
Takano et al. [57]	None	2; 12.6 months; 2 PR	None	None	None
Pao et al. [58]	None	1; 13 months; 1 PR	None	None	None
Total, n	44	127	71	32	4
ORR, n (%)	31 (70.5%)	68 (53.5%)	34 (47.9%)	10 (31.2%)	NA
mPFS, range (months)	2–17.5	1.9–24.5	1.1–12	1.6–10	6

*PFS* progression-free survival, *TKI* tyrosine kinase inhibitor, *mPFS* median progression-free survival, *PR* partial response, *NA* not available, *SD* stable disease, *PD* progressive disease, *CR* complete response, *SR* serological response, *ORR* overall response rate

^a^mPFS not reached

*19Del* and *L858R* mutations are classical sensitizing mutations, and the strong response of these two mutations to TKIs has been demonstrated in many prospective studies [2–5, 9]. However, these two common mutations are frequently detected concomitant with other mutations in the compound *EGFR*-mutated population. A compound mutation co-existing with a *19Del* or *L858R* mutation was the most common combination in previous reports (203/278, 73.0%) (Table 4) and in our cohort (85/106, 80.2%) (Table 3). Xu et al. [16] reported that tumours with double common *EGFR* mutations (*19Del *+* L858R*, *n* = 18) exhibited similar antitumour responses to small-molecule TKIs as tumours with single common mutations, and the median PFS and ORR rates were 9.53 months and 71.4% (10/14), respectively, which is consistent with our results (10.1 months and 60%, respectively) and those of Hata Akito (16.5 months and 86%, respectively) [11]. Some case reports also found that first-generation *EGFR* TKIs may be a desirable therapeutic strategy for patients with advanced lung cancer with synchronous *19Del* and *L858R* mutations [8, 9, 35].

In patients harbouring common plus rare mutation, the *L858R* mutation was more frequently observed than the *19Del* mutation. For example, approximately 10% and 17.3% of NSCLC patients harboured the L858R mutation concomitantly with rare mutations in the two cohorts reported by Wu et al. [17] and Kobayashi et al. [8], respectively. Similarly, in our common plus rare mutation subgroup, *L858R*, was identified in the majority of the cases (9/11, 81.8%).

The response to TKIs in patients with common plus rare mutations and whether TKI therapy prolonged PFS remains controversial because of the relatively large heterogeneity. Keam [14] reported an RR of 68.8% and median PFS time of 8.1 months in 16 patients, which are similar to our observed RR (45.5%) and median PFS time (10.5 months) in 11 patients. Notably, this above finding was also reported previously [16, 18]. As a whole, this population may benefit from TKIs, but to lesser extent than the population harbouring a single common mutation. We found that the patients with *L858R *+* K757R* mutations (exon 21 + exon 19) and *L858R* + *I744M* mutations (exon 21 + exon 19) exhibited a partial response to gefitinib and obtained PFS of 9.0- and 9.6-month, respectively. Klughammer et al. [19] and Kempf et al. [20] reported that a single *I744M* mutation or a single *K757R* mutation in exon 19 may be TKI-sensitizing mutations, and these mutations were also observed to have PR to oral TKI therapy. Therefore, the above double or single mutation(s) patterns may be candidates for TKI therapy. Patients with exon 20 mutations are considered resistant to TKIs (discussed below), but our study included a patient with *L858R* plus the *R776H* mutation (exon 21 + exon 20) showing PR to TKIs for 10.5 months, which is also highly consistent with prior reports [8, 21]. Other patients with the *L858R* mutation associated with *S720P*, *S768I*, *L858Q* or *L833V* were classified in the insensitive to TKIs group, and most (5/6, 83.3%) exhibited PD or SD to TKI therapy. To the best of our knowledge, our study is the first to report the combinations of *L858R *+* S720P* mutations (exon 21 + exon 18) and *L858R* +* L833V* mutations (exon 21 + exon 21). No patient with *L833V *+ *H835L* mutations (exon 21 + exon 21) was detected in our cohort; however, patients with this combination have been reported to have a good response to gefitinib [22, 23]. One case is especially notable. Leventakos et al. [24] demonstrated that patients with *L858R *+ *S768I* mutations may be sensitive, or at least not resistant, to TKI therapy, which is in contrast to our results. Our patients with a single *L861Q* mutation or compound mutations with *L861Q* exhibited a high RR (66%) and non-inferior PFS (median, 6 months) to TKI therapy. We also detected two cases with *19Del *+* L861Q* mutations, and a good response to TKIs (one PR and one SD) and prolonged PFS (11.4 and 11.9 months) were observed. However, the patient with *L858R* + *L861Q* exhibited PD after only 1 month of TKI therapy.

Notably, common mutations concomitant with an initial *T790M* mutation accounted for 65.1% of all compound mutations in our cohort, which was higher than in previous reports [13]. However, Su et al. [13] verified that pre-treatment of a co-existing *EGFR T790M* mutation was not a rare event (23/73, 31.5%), and the PFS was significantly shorter than that in patients without *T790M* (median, 6.7 months vs. 10.2 months, respectively; *P* = 0.035) [13]. *T790M* status also affected PFS in our cohort compared with a single-sensitizing *EGFR* mutation (9.1 months vs. 13.0 months, respectively; *P* < 0.001). We hypothesize that the scarcity of reports may be due to the bias of excluding patients with *T790M* [21]. Previous research on compound mutations may have overlooked the fact that—*T790M* may occur in patients before receiving *EGFR* TKI treatment [1, 21]. In addition, direct sequencing may be used to detect the classical sensitizing mutations in exons 19 and 21, which leads to a missed opportunity to discover patients with co-occurring *T790M*. The impact of *EGFR* TKIs in these patients with *19Del* or *L858R* plus *T790M* was not clarified because of the scarcity of patients and the varying durations of PFS in the published literature. However, approximately one-third and one-half of patients with concomitant initial *T790M* as one of the compound mutations in previous studies [13] and our cohort obtained more than 8 months of PFS with the aid of TKI therapy. Therefore, small molecule TKIs may be an optional therapeutic strategy to identify potential beneficiaries after explaining the bias of the therapy to patients in detail to ensure patient understanding and informed consent.

Patients who harbour a single exon 20 mutation in *EGFR* are reportedly insensitive to small-molecule TKIs [14, 19, 25–27]. However, whether patients with an *EGFR* exon 20 mutation accompanied by another mutation are candidates for TKI therapy remains unanswered. Marius Lund-Iversen and his colleagues reported seven exon 20-positive patients who received oral TKI, including five patients with single exon 20 mutation and two patients with double mutations. The five patients with single exon 20 mutation were found to have progressive disease at the first post-treatment follow-up, but the two patients with double mutations obtained 11 and 14 months of an ongoing response [28]. Chen et al. [18] also concluded that patients with compound mutations involving mutation in exon 20 benefited from TKIs more than single exon 20 mutations [18]. The duration of response to TKIs in compound *EGFR*-mutated patients with concomitant exon 20 mutation (excluding *T790M*) (6.5 months) was still shorter than compound mutated patients without exon 20 mutation (9.1 months) and patients with single common mutations (13.0 months), which is consistent with Keam et al. [14] (< 5 months). The analytical results of Kancha et al. [29] and Wu et al. [17] also support this finding. Together this suggests that first-generation *EGFR* TKIs may not be suitable for patients with an exon 20 mutation regardless of the presence of other mutations.

Overall, patients with double rare mutations or a rare mutation plus *T790M* exhibited a lower RR (38.5% and 37.5%, respectively) and worse PFS to TKI therapy (median, 6.5 and 5.4 months, respectively) in our cohort, which is consistent with a previous publication [14]. A patient with a single *L861Q* point mutation at exon 21 and a *G719X* point mutation at exon 18 may be classified into the TKI-sensitive mutation group [30]. However, patients with a *L861Q* or *G719X* mutation co-occurring with a rare mutation or *T790M* affected the effectiveness and sensitivity to TKI therapy in our clinical practice (RR, 28.6%; median PFS, 5.1 months). We found five patients with a rare mutation plus *T790M*, which is more than the overall number of reported cases. One patient with *G719X *+ *T790M* mutations and one patient with *L858Q *+ *T790M* mutations exhibited PR to TKI therapy and obtained more than 10 months PFS, which was similar to a case report from Chen et al. [18]. Balak et al. [31] found that the *D761Y* mutation in exon 19 was a novel secondary resistance mutation to *EGFR* TKIs [31]. Therefore, the two patients with two resistance mutations (*D761Y *+* T790M*) in our study exhibited disease progression very soon after initiating TKI therapy, which was not surprising.

The population in our study was a fairly large cohort to investigate the effectiveness of TKI therapy in patients with compound *EGFR*-mutated lung cancer. However, this study was a retrospective analysis, which may limit the reliability of the results. Potential selective bias may be unavoidable because of the low incidence of occurrence of these types of mutations. In addition, the RR and PFS of patients with compound *EGFR* mutations were compared only between patients who received *EGFR* TKI therapy without inclusion of patients who received chemotherapy. Our data were collected from a single institution, and patients from other areas of China should be examined. All reported cases between 2004 and 2017 were enrolled, but the literature from Asia still accounts for the majority of available data.

## Conclusions

Although NSCLC patients with compound mutations exhibited a shorter RFS and lower RR in response to TKI therapy than those with a single common mutation, TKI therapy may still benefit patients with compound mutations. Therefore, after explaining the biases of TKI therapy to patients in detail to ensure their understanding and informed consent, a trial of first-generation small molecule TKIs may be an optional therapeutic strategy to identify potential beneficiaries.

## Abbreviations

*EGFR*: epidermal growth factor receptor
TKIs: tyrosine kinase inhibitors
NSCLC: non-small cell lung cancer
PFS: progression-free survival
OS: overall survival
ADC: adenocarcinoma
SCC: squamous cell carcinoma
LELC: lymphoepithelioma-like carcinoma
ASC: adenosquamous carcinoma
PD: progressive disease
SD: stable disease
PR: partial response
CR: complete response
NE: not evaluated
